# Supplementary material for: Accessibility to rabies centers and human rabies post-exposure prophylaxis rates in Cambodia: A Bayesian spatio-temporal analysis to identify optimal locations for future centers
Source: PLoS Negl Trop Dis. 2022 Jun 30;16(6):e0010494. doi: 10.1371/journal.pntd.0010494 (PMC9491732; doi:10.1371/journal.pntd.0010494)
Supplement: S1 Table — (DOCX) [file pntd.0010494.s001.docx]

***S1 Table:*** ***Observed cumulative PEP patients by province from 2000 to 2016.***

| Province | PEP patients | PEP rate  per 10,000  person-year |
| --- | --- | --- |
| KH01 Banteay Mean Chey | 229 | 0.19 |
| KH02 Battambang | 507 | 0.31 |
| KH03 Kampong Cham | 22,809 | 8.09 |
| KH04 Kampong Chhnang | 4,143 | 5.19 |
| KH05 Kampong Speu | 9,870 | 8.05 |
| KH06 Kampong Thom | 3,432 | 3.22 |
| KH07 Kampot | 4,465 | 4.57 |
| KH08 Kandal | 60,267 | 29.30 |
| KH09 Koh Kong | 291 | 1.45 |
| KH10 Kratie | 545 | 1.01 |
| KH11 Mondul Kiri | 46 | 0.45 |
| KH12 Phnom Penh | 158,009 | 65.33 |
| KH13 Preah Vihear | 67 | 0.23 |
| KH14 Prey Veaeng | 12,507 | 7.60 |
| KH15 Pursat | 435 | 0.65 |
| KH16 Ratanakiri | 66 | 0.26 |
| KH17 Siem Reap | 356 | 0.24 |
| KH18 Preah Sihanouk | 482 | 1.35 |
| KH19 Stueng Treng | 40 | 0.21 |
| KH20 Svay Rieng | 1,738 | 2.09 |
| KH21 Takeo | 13,563 | 9.47 |
| KH22 Otdar Meanchey | 42 | 0.14 |
| KH23 Kep | 12 | 0.20 |
| KH24 Pailin | 34 | 0.33 |
