# Supplementary material for: Accessibility to rabies centers and human rabies post-exposure prophylaxis rates in Cambodia: A Bayesian spatio-temporal analysis to identify optimal locations for future centers
Source: PLoS Negl Trop Dis. 2022 Jun 30;16(6):e0010494. doi: 10.1371/journal.pntd.0010494 (PMC9491732; doi:10.1371/journal.pntd.0010494)
Supplement: S2 Table — Scenario 2 represents the current situation in Cambodia with three PEP centers available in Phnom Penh, Battambang and Kampong Cham. Scenario 5 includes 21 models that each add one center from the 21 remaining provinces to the three in existence. Scenario 5 was used to identify the best future location for adding new centers. (DOCX) [file pntd.0010494.s002.docx]

***S2 Table: Predicted accessibility to PEP centers and new PEP patients in the 21 simulations of Scenario 5 in comparison with Scenario 2.***

*Scenario 2 represents the current situation in Cambodia with three PEP centers available in Phnom Penh, Battambang and Kampong Cham. Scenario 5 includes 21 models that each add one center from the 21 remaining provinces to the three in existence. Scenario 5 was used to identify the best future location for adding new centers.*

|  | Population in districts  with a median travel time  to a PEP center below 60min | Difference  in population  from Scenario 2 | Predicted  patients | Difference  in PEP patients  from Scenario 2 |
| --- | --- | --- | --- | --- |
| Scenario 2 | 5,386,549 | NA | 29,950 | NA |
| KH01 Banteay Mean Chey | 5,889,612 | 503,063 | 32,494 | 2,544 |
| KH04 Kampong Chhnang | 5,584,194 | 197,645 | 31,158 | 1,208 |
| KH05 Kampong Speu | 5,488,476 | 101,927 | 31,546 | 1,596 |
| KH06 Kampong Thom | 5,529,896 | 143,347 | 31,591 | 1,641 |
| KH07 Kampot | 5,767,358 | 380,809 | 31,768 | 1,818 |
| KH08 Kandal | 5,386,549 | 0 | 31,062 | 1,112 |
| KH09 Koh Kong | 5,414,599 | 28,050 | 30,240 | 290 |
| KH10 Kratie | 5,477,115 | 90,566 | 31,066 | 1,116 |
| KH11 Mondul Kiri | 5,399,510 | 12,961 | 30,143 | 193 |
| KH13 Preah Vihear | 5,422,345 | 35,796 | 30,509 | 559 |
| KH14 Prey Veaeng | 5,704,213 | 317,664 | 31,242 | 1,292 |
| KH15 Pursat | 5,749,240 | 362,691 | 31,034 | 1,084 |
| KH16 Ratanakiri | 5,439,867 | 53,318 | 30,518 | 568 |
| KH17 Siem Reap | 6,053,755 | 667,206 | 33,286 | 3,336 |
| KH18 Preah Sihanouk | 5,512,641 | 126,092 | 30,784 | 834 |
| KH19 Stueng Treng | 5,421,975 | 35,426 | 30,457 | 507 |
| KH20 Svay Rieng | 6,069,565 | 683,016 | 32,300 | 2,350 |
| KH21 Takeo | 5,890,556 | 504,007 | 32,376 | 2,426 |
| KH22 Otdar Meanchey | 5,452,168 | 65,619 | 30,760 | 810 |
| KH23 Kep | 5,601,545 | 214,996 | 31,231 | 1,281 |
| KH24 Pailin | 5,458,289 | 71,740 | 30,528 | 578 |
