# Supplementary material for: Accessibility to rabies centers and human rabies post-exposure prophylaxis rates in Cambodia: A Bayesian spatio-temporal analysis to identify optimal locations for future centers
Source: PLoS Negl Trop Dis. 2022 Jun 30;16(6):e0010494. doi: 10.1371/journal.pntd.0010494 (PMC9491732; doi:10.1371/journal.pntd.0010494)
Supplement: S3 Table — Results from both province and district level models are presented. Scenario 1 represents the situation prior to the opening of new centers in Battambang and Kampong Cham provinces with a single center in Phnom Penh. Scenario 2 represents the current situation, with the opening of two new centers in Battambang and Kampong Cham provinces that actually opened in 2018 and 2019 respectively, bringing the total number of centers to three. Scenario 3 represents the theoretical opening of a center in every provincial capital. Scenario 4 represents the theoretical opening of a center in every district. (DOCX) [file pntd.0010494.s003.docx]

***S3 Table:*** ***Nation-wide predictions of PEP patients numbers and rates according to four prediction scenarios.***

*Results from both province and district level models are presented. Scenario 1 represents the situation prior to the opening of new centers in Battambang and Kampong Cham provinces with a single center in Phnom Penh. Scenario 2 represents the current situation, with the opening of two new centers in Battambang and Kampong Cham provinces that actually opened in 2018 and 2019 respectively, bringing the total number of centers to three. Scenario 3 represents the theoretical opening of a center in every provincial capital. Scenario 4 represents the theoretical opening of a center in every district.*

| Measure | Scenario | province level  numbers | province level  rates | district level  numbers | district level  rates |
| --- | --- | --- | --- | --- | --- |
| PEP patients  (rate per  10,000 people) | 2016 observed | 21,611 |  | 21,611 |  |
|  | 2016 fitted values | 21,609 (21,290 to 21,932) | 14.02 (14.49 to 14.92) | 21,623 (21,259 to 22,002) | 14.71 (14.46 to 14.97) |
|  | 2017 (scenario 1) | 21,885 (17,477 to 27,440) | 14.73 (11.78 to 18.43) | 21,712 (20,471 to 23,048) | 14.61 (13.77 to 15.51) |
|  | 2017 (scenario 2) | 28,040 (22,425 to 35,095) | 18.87 (15.09 to 23.62) | 29,950 (28,235 to 31,819) | 20.15 (19.00 to 21.41) |
|  | 2017 (scenario 3) | 42,992 (34,365 to 53,825) | 28.93 (23.12 to 36.22) | 50,944 (48,012 to 54,128) | 34.28 (32.31 to 36.42) |
|  | 2017 (scenario 4) | 76,638 (61,206 to 96,046) | 51.57 (41.19 to 64.63) | 92,601 (86,899 to 98,789) | 62.31 (58.47 to 66.47) |
