# Supplementary material for: Accessibility to rabies centers and human rabies post-exposure prophylaxis rates in Cambodia: A Bayesian spatio-temporal analysis to identify optimal locations for future centers
Source: PLoS Negl Trop Dis. 2022 Jun 30;16(6):e0010494. doi: 10.1371/journal.pntd.0010494 (PMC9491732; doi:10.1371/journal.pntd.0010494)
Supplement: S4 Table — Results from both province and district level models are presented. Scenario 1 assumes no opening of new vaccination centers. Scenario 2 assumes the opening of two new centers in Battambang and Kampong Cham provinces that actually opened in 2018 and 2019 respectively. Scenario 3 assumes the opening of a center in every provincial capital. Scenario 4 assumes the location of a vaccination center in every district. This is a theoretical scenario used as a proxy for universal access. (DOCX) [file pntd.0010494.s004.docx]

***S4 Table:*** ***Predicted number of patients by province based on four prediction scenarios.***

*Results from both province and district level models are presented, with district results aggregated to provinces. These are compared to the observed data from 2016. Scenario 1 assumes no opening of new vaccination centers. Scenario 2 assumes the opening of two new centers in Battambang and Kampong Cham provinces that actually opened in 2018 and 2019 respectively. Scenario 3 assumes the opening of a center in every provincial capital. Scenario 4 assumes the location of a vaccination center in every district. This is a theoretical scenario used as a proxy for universal access.*

| Provinces | Province level model | | | | | District level model | | | | |
| --- | --- | --- | --- | --- | --- | --- | --- | --- | --- | --- |
|  | 2016 | 2017-I | 2017-II | 2017-III | 2017-IV | 2016 | 2017-I | 2017-II | 2017-III | 2017-IV |
| KH01 Banteay Mean Chey | 11 | 19 | 972 | 2248 | 3951 | 19 | 7 | 1055 | 2819 | 4589 |
| KH02 Battambang | 26 | 34 | 1472 | 2131 | 5324 | 58 | 31 | 2515 | 2948 | 6104 |
| KH03 Kampong Cham | 1751 | 1033 | 3818 | 3914 | 8404 | 1677 | 1290 | 2942 | 2963 | 6122 |
| KH04 Kampong Chhnang | 380 | 546 | 547 | 1146 | 2434 | 417 | 722 | 724 | 1709 | 2880 |
| KH05 Kampong Speu | 873 | 1021 | 1021 | 1968 | 3879 | 903 | 1638 | 1638 | 2931 | 5046 |
| KH06 Kampong Thom | 235 | 177 | 263 | 1075 | 2789 | 255 | 253 | 433 | 1658 | 3735 |
| KH07 Kampot | 256 | 333 | 333 | 1449 | 2694 | 278 | 332 | 332 | 1832 | 3524 |
| KH08 Kandal | 4213 | 3170 | 3170 | 3520 | 5312 | 4391 | 3681 | 3681 | 4311 | 7569 |
| KH09 Koh Kong | 14 | 4 | 4 | 119 | 460 | 10 | 16 | 16 | 246 | 753 |
| KH10 Kratie | 31 | 14 | 67 | 703 | 1133 | 38 | 14 | 109 | 982 | 1703 |
| KH11 Mondul Kiri | 4 | 0 | 1 | 72 | 167 | 2 | 0 | 2 | 114 | 206 |
| KH12 Phnom Penh | 11260 | 13277 | 13277 | 13844 | 15440 | 10622 | 10671 | 10671 | 10919 | 12473 |
| KH13 Preah Vihear | 8 | 2 | 3 | 297 | 1175 | 7 | 1 | 3 | 411 | 1812 |
| KH14 Prey Veaeng | 1303 | 1011 | 1435 | 2444 | 5247 | 1399 | 1145 | 1546 | 3216 | 7513 |
| KH15 Pursat | 33 | 42 | 141 | 483 | 1656 | 48 | 76 | 269 | 1150 | 1696 |
| KH16 Ratanakiri | 10 | 0 | 0 | 262 | 918 | 8 | 0 | 0 | 462 | 1159 |
| KH17 Siem Reap | 23 | 49 | 290 | 2181 | 5419 | 33 | 31 | 291 | 3276 | 5890 |
| KH18 Preah Sihanouk | 31 | 103 | 103 | 662 | 1298 | 29 | 45 | 45 | 682 | 1083 |
| KH19 Stueng Treng | 3 | 0 | 2 | 177 | 425 | 4 | 0 | 1 | 289 | 423 |
| KH20 Svay Rieng | 191 | 244 | 280 | 1396 | 2049 | 226 | 233 | 270 | 1857 | 2993 |
| KH21 Takeo | 946 | 794 | 794 | 2092 | 4575 | 1017 | 1213 | 1213 | 2891 | 6650 |
| KH22 Otdar Meanchey | 1 | 1 | 13 | 448 | 1445 | 2 | 0 | 10 | 571 | 2919 |
| KH23 Kep | 5 | 11 | 11 | 147 | 164 | 9 | 8 | 8 | 185 | 208 |
| KH24 Pailin | 3 | 0 | 23 | 214 | 280 | 5 | 0 | 15 | 241 | 303 |
| KH25 Tbong Khmum | NA | NA | NA | NA | NA | 186 | 305 | 2161 | 2281 | 5248 |
