# Supplementary figures and images for: Accessibility to rabies centers and human rabies post-exposure prophylaxis rates in Cambodia: A Bayesian spatio-temporal analysis to identify optimal locations for future centers
Source: PLoS Negl Trop Dis. 2022 Jun 30;16(6):e0010494. doi: 10.1371/journal.pntd.0010494 (PMC9491732; doi:10.1371/journal.pntd.0010494)

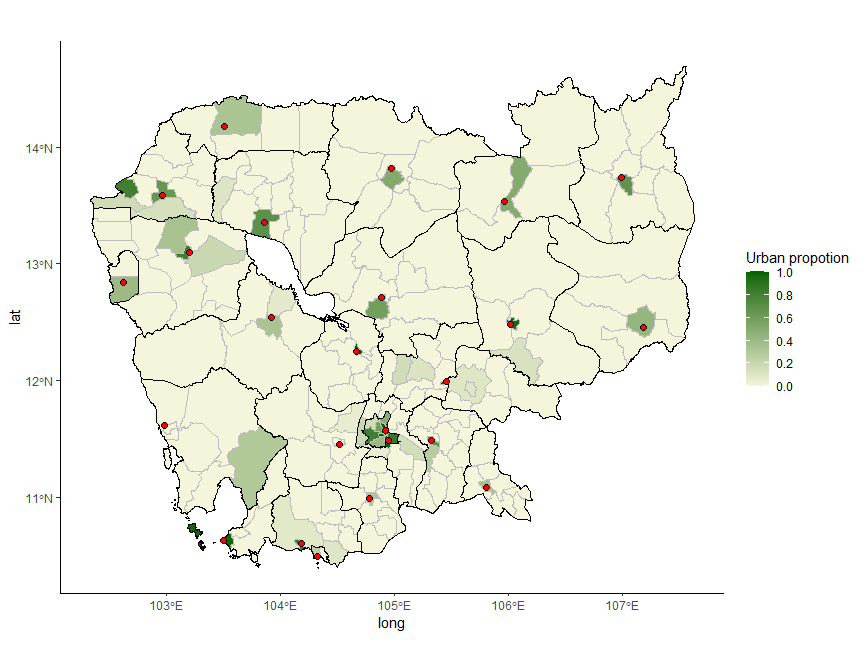

Supplement: S1 Fig — Estimates based on linear projections of the urban and rural populations by district from the 1998 and 2008 census. Red dots represent the location of provincial capitals. Most urbanized districts in Cambodia are where provincial capitals are located. Base map can be found at [45]. https://data.humdata.org/dataset/cambodia-admin-level-0-international-boundaries. Details for the corresponding license can be found at: https://data.humdata.org/faqs/licenses. (TIF) [file pntd.0010494.s005.tif]

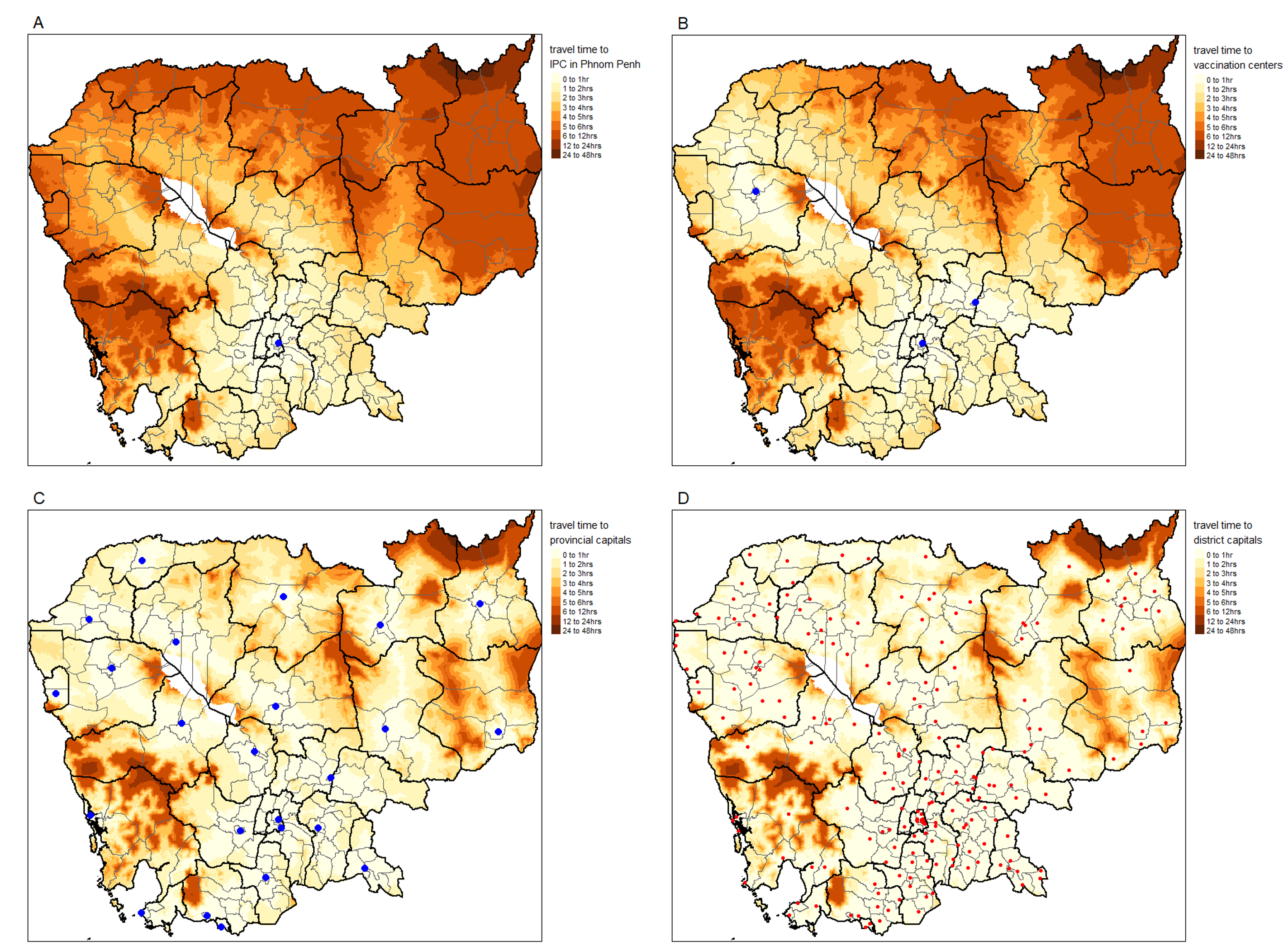

Supplement: S2 Fig — Travel time to point rasters for (A) IPC in Phnom Penh as was the case up to 2017, (B) all three current vaccination centers including the one in Battmabang opened in 2018 and the one in Kampong Cham opened in 2019,(C) all provincial capitals in Cambodia based on the 2010 administrative break-down and (D) all district capitals. Blue dots represent the points of interest for each maps: vaccination centers or provincial capitals. Base map can be found at [46]. https://gadm.org/download_country.html. Details for the corresponding license can be found at: https://gadm.org/license.html. (TIF) [file pntd.0010494.s006.tif]

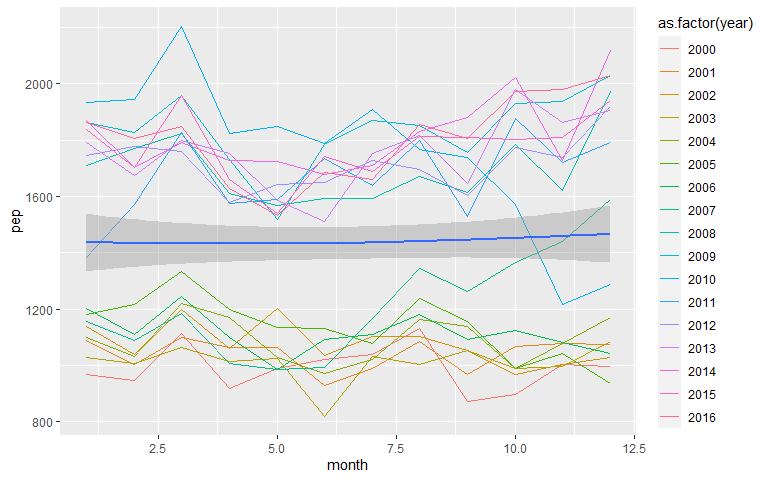

Supplement: S3 Fig — GAM curve is represented with a thick blue line and uncertainty shading. (TIF) [file pntd.0010494.s007.tif]
