# Supplementary material for: Accessibility to rabies centers and human rabies post-exposure prophylaxis rates in Cambodia: A Bayesian spatio-temporal analysis to identify optimal locations for future centers
Source: PLoS Negl Trop Dis. 2022 Jun 30;16(6):e0010494. doi: 10.1371/journal.pntd.0010494 (PMC9491732; doi:10.1371/journal.pntd.0010494)
Supplement: Attachment — Submitted filename: PNTD-D-21-00896.pdf [file pntd.0010494.s011.pdf]

Thank you for the opportunity to review this interesting paper on the effect of accessibility to PEP providing health centers on bite patient estimates to support rabies control strategies. The authors have written a very good piece and the scope of the work fits well with the PLoS NTD audience. Rabies research faces a lot of challenges regarding data availability and the authors applied thorough methods to estimate the number of actual bite victims in comparison to the reported bite victims in Cambodia. However, some of the methods and results have to be adapted to further improve the validity of the outcomes.

My main concerns are related to the population data in combination with the accessibility measures. Aggregated population counts in combination with aggregated measures of accessibility, create more uncertainty in the predictions and outcomes mentioned in the results. Since the travel time rasters are gridded I would highly suggest the authors to also use a gridded population dataset that provides information on the location of the population, like the WorldPop constrained dataset.

In addition, the way the results are written make it hard to follow the actual goal of the paper, which in my opinion is to estimate the risk of a rabid animal bite. I would consider a stronger emphasis on the number of patients estimated to be missed by the PEP clinics (estimated patients – reported patients) and the number of estimated rabid positive bites (number of positive samples * estimated animal bites). A visual representing the aimed outcomes or a decision tree model would maybe help guide the reader in understanding the results better. I provide more detail below.

The paper would also greatly benefit from a detailed read through to improve grammar. There are quite some inconsistencies with missing words or words that are plural and miss and s at the end.

Finally, the accessibility measures that are adapted from the global friction surfaces as provided by the Malaria project introduce uncertainty on actual travel times of the target population, because speeds on roads and landcover types have been set-up in a global studuy, not always reflecting the local context realistically.

Yet, the paper provides useful insights into an important NTD in a context that lacks recent research. The paper has high potential for publication when the feedback provided is taken into consideration.

All the best with the suggestions and I’m looking forward to reading an improved version of the paper!

<u>Abstract:</u>
The abstract is well written, but I think that the explanation of the most important definitions such as “risk” and “PEP patients” is missing. This could be easily changed by indicating the exact risk, for example risk of dogbites or risk of rabies. This is the same for PEP patients, by indicating whether it’s the number of dogbite patients receiving PEP or something else. I would also suggest a stronger emphasis on the outcomes of the Bayesian models, so that the risk and human burden is also highlighted in the abstract.

I would suggest to call PEP patients either dogbite or animal bite patients, or explain once "dogbite patients, from now on referred to as PEP patients".

In addition, there are frequently some words missing. I will highlight them below.

Line 30: the word "it" misses. Suggest changing to "making it difficult"
Line 30: I would rewrite "making it difficult to establish a human burden of disease and generate a risk map" to "making it difficult to estimate/model the human burden of disease and generate a risk map of dog bites/rabies"
Line 34: Clarify the outcome variable and add "s" to read "PEP patients as outcomes". Is the outcome variable the number of patients seeking PEP treatment?
Line 35: I would suggest changing to "The primary (exposure) variable"
Line 35-36: I would suggest changing to "The secondary (exposure) variable"
Line 39: remove the word "in" so sentence reads "an increase in one hour travel time leads to a.."

Author summary:
Line 49: "Rabies is a fatal"
Line 52: "is highly effective at preventing the disease"

Introduction:
The introduction is well written and provides a strong background of the current global and Cambodian rabies situation. Very well done! Find some little suggestions for improvement below.

Line 107: change suspect to suspected so it reads **"**suspected animals responsible.."
Line 127-128: This sentence is grammatically a bit difficult to read because "and" is used twice. I would also suggest introducing accessibility as either geographical, spatial, or physical accessibility and then say "from now on referred to as accessibility" because there are different types of access.
Line 136: change "to Cambodian population" to " to the Cambodian population"

Methods
The methods chosen seem to have been carefully considered and I support the decision to use an INLA model, because of the widely available options to incorporate spatial and temporal dependency of the data. The authors managed to explain the four different scenarios very well and have scientifically sounds approach. However, I feel that the description is not always clear and the objectives become a little bit fuzzy after the very clear introduction. If indeed the goal of this paper is to estimate the spatial distribution of the risk of dogbite among all provinces and districts of Cambodia, then the explanation of the methods would have to be restructured.

I would consider merging the estimates of dogbite patients and the estimates of positive animal samples so that you can say something about the proportion of rabid dogbites among people bringing in a sample along with their need for PEP.

In addition, the methods lack an explanation on data cleaning or incomplete bite reports. I would expect that not all patients were bitten by dogs or animals capable of transmitting rabies, so this raises the question how many reports had to be removed because of that. In addition, I was wondering how many bite reports couldn't be linked to the right administrative boundaries because of incompleteness or misspelling. Maybe this could be elaborated in the Supplementary material.

Population data is the main denominator for the outcomes of your estimates, make sure to use one that is reliable across all years. I would also suggest comparing the estimates made in this study to the widely available open datasets from for instance WorldPop or the CIESN/facebook team. This would give you more precise input data about the location of your data for the eventual demographic estimates. I would also suggest you to have a look at the R package wopr (https://github.com/wpgp/wopr) to support the estimates you make for the population data as described in lines 162-165. Find below some additional points of feedback:

Line 216-218: "The accessibility rasters were aggregated for each province and district polygon by extracting the median travel time from patient residence province or district to PEP center or provincial capital value."

This sentence is a bit hard to follow, I would suggest rewriting to: The accessibility measure was aggregated by means of extracting the median travel time per province or district. This could ultimately be linked to the patient's record (i.e. the district or province of residence).

Paragraph 2.2.1: I would advise you to use a constrained (using building footprints) gridded population dataset as input data (https://www.worldpop.org/methods/top_down_constrained_vs_unconstrained). It allows you to have more accurate information on the actual location of population and thus the population living beyond the travel time catchments you are indicating (i.e 60 and 120 min). The calculations you make involve two aggregated measures (namely aggregated travel time and aggregated population counts) while in fact you have finer scale spatial data available to deal with the calculations in a more precise manner.

Paragraph 2.2.3: This paragraph is not very clear to me and my feedback links back to the more general point raised before "to consider merging the estimates of dogbite patients and the estimates of positive animal samples". To me it does not seem logical to predict the number of dogs that would be brought in for testing. This would make sense if your goal is to scale up your FAT testing capacity, but not to predict the risk of rabid dogbites. In my opinion it would make more sense if you use the number of positively tested samples among the patients that brought in a sample, as a proxy for the number of rabid dog bites and thus potentially averted deaths by the use of PEP. In a recent article written by Rajeev et al (2021), dogbites in Madagascar were used in a decision tree model to estimate the risk of a rabid dogbite in a Bayesian MCMC model. If this is also your aim, I would consider redoing a part of your model here and re-describe your goal and method here.

Line 252-254: Sentence "In an aggregated logistic regression.." is a bit challenging to read I would suggest to rewrite.

<u>Results</u>
The results presented reflect the methods introduced before and are clearly lined out in text and figures. However, I find paragraph 3.3. challenging and would suggest to restructure and rewrite this. Below you can find a more detailed description.

Line 279-280: Were all these patient reports complete? How many were available at the province level and how many at the district level?

Paragraph 3.2: For the statistics about access to PEP I would highly suggest you to use a gridded population dataset instead of the aggregated counts. See example Rajeev et al. (2021). This would really increase the validity of your estimates. Proportionally calculating people's access to care without taking into account their exact location leads to too much uncertainty of your estimates.

Line 355-356: Can you compare the provincial and district models? Since not all input data was collected at the district level, I would also expect a less powerful model output on district level because not all bite reports can be brought back to this level.

Paragraph 3.3: It's a well written paragraph but it remains unclear to me what exactly you want to model and to communicate to your audience. I think the main goal is to understand the distribution of rabies risk and thus the need to improve the spatial availability of PEP in high risk regions. To me it seems therefore that you would like to report 1) on the number of patients estimated to have missed out on PEP because of poor access, which would be your predicted number of patients minus the reported number of animal bite patients 2) the estimated risk of a patient being bitten by a rabid animal, which would be the probability of an animal to be rabid times the number of reported animal bites.

The increase in PEP patients to me seems a bit as an indirect measure. Because it represents the estimated total number of patients based on access to PEP centers over the last years. So reporting onthe regions where the difference between reported and predicted bite victims is highest would be the most important outcome, that is where you are missing access.

<u>Discussion & conclusion</u>
Again, well written and provides a good overview of the results but would be more tangible with reports on measures as "averted deaths", "human burden of diseases", "proportion of rabid positive bites"

Line 431: "bite injuries" instead of "bites injuries"

Paragraph 4.2: The authors mention an important limitation of the accessibility layer used in paragraph 4.4, reflecting the static usage of the layer which is likely to have changed over the years because of improvements in the road network. However, the way accessibility is measured in this study brings another uncertainty to the estimates. The widely used malaria atlas friction layer makes large scale assumptions on road networks and travel speeds on different landcover types, which in fact might be different for your target population,

namely people who seek access to care. In the paper published by Weiss et al (2018) https://www.nature.com/articles/nature25181. You can find back the travel speeds applied to each country. I would highly suggest you to have a look and check whether they seem reliable for your target population.

Figures:
Overall great figures, but would suggest to clip the estimated travel times to the country borders.
