# Supplementary material for: Accessibility to rabies centers and human rabies post-exposure prophylaxis rates in Cambodia: A Bayesian spatio-temporal analysis to identify optimal locations for future centers
Source: PLoS Negl Trop Dis. 2022 Jun 30;16(6):e0010494. doi: 10.1371/journal.pntd.0010494 (PMC9491732; doi:10.1371/journal.pntd.0010494)
Supplement: Attachment — Submitted filename: Response to reviewers.docx [file pntd.0010494.s012.docx]

| UNIVERSITY OF CALIFORNIA, DAVIS | |  |  | |
| --- | --- | --- | --- | --- |
| BERKELEY • DAVIS • IRVINE • LOS ANGELES • MERCED • RIVERSIDE • SAN DIEGO • SAN FRANCISCO | |  | SANTA BARBARA • SANTA CRUZ | |
| UNIVERSITY OF CALIFORNIA  Center for Animal Disease Modeling and Surveillance (CADMS)  Department of Medicine & Epidemiology  School of Veterinary Medicine  Phone: (530) 752-7675  Fax: (530) 752-0414 | ONE SHIELDS AVENUE  DAVIS, CALIFORNIA 95616-8734 | | |  |

Uppsala, Sweden, November 5, 2021

Dear editors and reviewers

We thank you for your comments and suggestions, and for extending the submission deadline on re-submitting this paper. Please find attached our response to the reviewers’ comments to our manuscript entitled “Impact of accessibility to rabies centers on human rabies post-exposure prophylaxis rates in Cambodia and identification of optimal future center locations using spatio-temporal Bayesian regression modelling”, as well as an edited copy taking into account these comments. We hope we have addressed the comments as to make this manuscript suitable for publication.

Thank you in advance for your consideration,

Best Regards,

Jerome Baron, DVM, MSc

Center for Animal Disease Modeling and Surveillance (CADMS)

Department of Medicine & Epidemiology

School of Veterinary Medicine

University of California

Davis, CA 95616 USA

jnbaron@ucdavis.edu

Section for Epidemiological Methods

Department of Epidemiology and Disease Control
National Veterinary Institute of Sweden

Uppsala, 75189 Sweden

jerome.baron@sva.se

Reviewer's Responses to Questions

**Key Review Criteria Required for Acceptance?**


As you describe the new analyses required for acceptance, please consider the following:

**Abstract:**

The abstract is well written, but I think that the explanation of the most important

definitions such as “risk” and “PEP patients” is missing. This could be easily changed by

indicating the exact risk, for example risk of dogbites or risk of rabies. This is the same for

PEP patients, by indicating whether it’s the number of dogbite patients receiving PEP or

something else. I would also suggest a stronger emphasis on the outcomes of the Bayesian

models, so that the risk and human burden is also highlighted in the abstract.

I would suggest to call PEP patients either dogbite or animal bite patients, or explain once

“dogbite patients, from now on referred to as PEP patients”.

In addition, there are frequently some words missing. I will highlight them below.

**Line 30:** the word “it” misses. Suggest changing to “making it difficult”

corrected

**Line 30:** I would rewrite “making it difficult to establish a human burden of disease and

generate a risk map” to “making it difficult to estimate/model the human burden of disease

and generate a risk map of dog bites/rabies”

corrected as suggested

**Line 34:** Clarify the outcome variable and add “s” to read “PEP patients as outcomes”. Is the

outcome variable the number of patients seeking PEP treatment?

corrected

**Line 35:** I would suggest changing to “The primary (exposure) variable”

corrected

**Line 35-36:** I would suggest changing to “The secondary (exposure) variable”

corrected

**Line 39:** remove the word “in” so sentence reads “an increase in one hour travel time leads

to a..”

corrected

**Author summary:**

**Line 49:** “Rabies is a fatal”

corrected

**Line 52:** “is highly effective at preventing the disease”

corrected

**Introduction:**

The introduction is well written and provides a strong background of the current global and

Cambodian rabies situation. Very well done! Find some little suggestions for improvement

below.

**Line 107:** change suspect to suspected so it reads **“**suspected animals responsible..”

corrected

**Line 127-128:** This sentence is grammatically a bit difficult to read because “and” is used

twice. I would also suggest introducing accessibility as either geographical, spatial, or

physical accessibility and then say “from now on referred to as accessibility” because there

are different types of access.

Modified as suggested

**Line 136:** change “to Cambodian population” to “ to the Cambodian population”
corrected

**Methods**


-Are the objectives of the study clearly articulated with a clear testable hypothesis stated?

-Is the study design appropriate to address the stated objectives?

-Is the population clearly described and appropriate for the hypothesis being tested?

-Is the sample size sufficient to ensure adequate power to address the hypothesis being tested?

-Were correct statistical analysis used to support conclusions?

-Are there concerns about ethical or regulatory requirements being met?


Reviewer #1: In this manuscript, the authors investigate the relationship between travel time to rabies treatment centers and the rate that individuals receive post-exposure prophylaxis (PEP) to prevent rabies in Cambodia. They first use a Bayesian regression model to fit the relationship between travel time and PEP rate using data reported from 2000-2016. They find an inverse relationship between travel time (accessibility) and PEP rate, as expected. Then they use this fitted relationship to estimate how much PEP would increase if additional testing facilities had been built in each province or district in 2017 (under 3 different scenarios). They conclude by identifying the 5 provincial capitals where they would recommend adding an additional treatment center.

What I liked about the manuscript was the creative approach the authors use to identify high-risk areas in the absence of reliable human disease risk or burden data. There are also several aspects of the rationale and methods that need to be addressed or clarified before publication, which I have described below.

1) Throughout the manuscript it was hard to follow what the authors meant by the different scenarios, and which ones they were referring to (table ST2 helped). I suggest (1) including at least one main table or diagram that describes the analytical approach clearly and (2) being explicit throughout the results and discussion section about which scenario and model (district or province) you are referring to. It would also be helpful to explicitly describe which model/scenario is used to recommend new treatment centers (and why that one was chosen).

A table describing the different prediction scenarios, and how they were used in the models was added (table 1). The scenario names were clarified in the results and discussion sections.

2) It would be helpful to have more details on how new treatment centers were placed in scenarios 2-4. In scenario 3 and 4, were the centers from scenario 2 that currently exist included (it appears this way from the figures)? In scenario 4, were the centers randomly place or were they placed within the largest city or existing health facilities/clinics? Do lines 215-216 refer to scenario 3, and was this the scenario used for final conclusions? Please clarify these points in the text.

Details on the placement of centers in all four scenarios were included in table 1. The models in lines 215-216 were renamed scenario 5 for clarity, with reference to how they differ from scenario 3. Description of their use for the final conclusions was added.

3) Why were Bayesian regression models chosen for identifying locations for a new treatment center? Since the goal is reducing travel time, why not just put a new center in a location that would maximally reduce travel time in either total hours or population-weighted in person-hours? Would this change the results? It looks like this might be what was done in “Section 3.2 Population accessibility”, but that section includes no corresponding methods or figures so it’s hard to follow.

Administrative centers were chosen as the location for theoretical new PEP centers as these are where they would most likely be introduced. However as these are also the main population and transportation centers of their spatial unit they most likely already maximally reduce population-weighted travel time. Given our spatial resolution, it is unlikely that testing and identifying specific locations within a city would have a major impact on aggregated accessibility values and thus model results. The corresponding methods to section 3.2 are described in section “2.2.1 Accessibility descriptive analysis”

4) What was the purpose of estimating the number of animals tested? This analysis doesn’t directly address the objectives of the study and isn’t included in the discussion. I suggest either removing it or making the rationale for doing it very clear (and then also including it in the discussion and conclusions).

This section was removed. Another paper focusing on dog testing will use some of these results.

5) On lines 517-520 you mention that there was no evidence for seasonality. Can you show this data in an SI figure or include a reference? You could also consider explicitly fit a non-linear model to confirm that seasonality isn’t captured here, and/or consider including a metric of seasonality in a model to test this (for example, including a monthly rainfall fixed effect). Though, I do not expect this to impact the overall conclusions.

A graph showing the monthly distribution of patients was added as well as the results of a non-linear model (gam) both to the text and graph.

Reviewer #2: Methods
The methods chosen seem to have been carefully considered and I support the decision to use an INLA model, because of the widely available options to incorporate spatial and temporal dependency of the data. The authors managed to explain the four different scenarios very well and have scientifically sounds approach. However, I feel that the description is not always clear and the objectives become a little bit fuzzy after the very clear introduction. If indeed the goal of this paper is to estimate the spatial distribution of the risk of dogbite among all provinces and districts of Cambodia, then the explanation of the methods would have to be restructured.

I would consider merging the estimates of dogbite patients and the estimates of positive animal samples so that you can say something about the proportion of rabid dogbites among people bringing in a sample along with their need for PEP.

Following a comment from reviewer one we chose to remove the section of the paper that focuses on dog testing as the main objective of this paper was to evaluate the impact of travel time on the number of PEP patients. We are working on a second paper focusing specifically predictors of rabies-positive test results amongst tested animals as a way to infer what proportion of patients were actually exposed to a rabid animal.

In addition, the methods lack an explanation on data cleaning or incomplete bite reports. I would expect that not all patients were bitten by dogs or animals capable of transmitting rabies, so this raises the question how many reports had to be removed because of that. In addition, I was wondering how many bite reports couldn’t be linked to the right administrative boundaries because of incompleteness or misspelling. Maybe this could be elaborated in the Supplementary material.

For the sake of this paper, we did not remove any patient report (unless location was not identifiable) as we were not looking specifically for confirmation of rabies exposure. The species of the animal and the confirmation of the rabies status in regards to that is explored in the second paper mentioned above. Only a small portion of patient records did not have an identified location and these are now mentioned in results section “3.1 descriptive data” .

Population data is the main denominator for the outcomes of your estimates, make sure to use one that is reliable across all years. I would also suggest comparing the estimates made in this study to the widely available open datasets from for instance WorldPop or the CIESN/facebook team. This would give you more precise input data about the location of your data for the eventual demographic estimates. I would also suggest you to have a look at the R package wopr (https://github.com/wpgp/wopr) to support the estimates you make for the population data as described in lines 162-165. Find below some additional points of feedback:

We compared our population projections to those of WorldPop and chose to retain our projections due to several considerations:

1) Since our initial submission, the full 2019 population census of Cambodia, including district level data, was released which made our population estimates much more robust. We thus re-ran all models with new and more accurate population estimates. Final results did not change much from the initial results.
2) We compared our population estimates to Worldpop data by aggregating the Worlpop data at the province level. From 2000 to 2008 the two data sources where nearly identical (as can be seen in the graphs below), but then deviated up to 2019 (when the most recent census was completed). However Worldpop metadata mentioned that its projections relied on the 1998 and 2008 census and then projected in the future without another datapoint after 2008. Thus within these two datapoints we observed very similar estimates between worldpop and our estimates but a divergeance once worldpop did not have an end data point for its projections, resulting in a much higher population estimated for Cambodia as whole than was actually measured in the 2019 cenus.
3) As our main outcome (PEP cases) was aggregated at the province or district level, the benefits of a higher resolution in the population data were deemed smaller than the drawbacks of the inaccuracies of worldpop projections in the more recent years.
4) However, as a sensitivity analysis effort, we did re-run all models using worldpop data as well and found that though there were some numerical differences in results it did not change the overall interpretation of these as can be seem in the table of fixed effect estimates below.
5) We investigated the R package wopr, but it seems this only focused on African population estimates and did not include data for Cambodia.

Graphs: comparison of province totals between census data and WorldPop estimates in the years 2000, 2008 and 2016. The 4^th^ graph just compares yearly total population in Cambodia using WorldPop projections (in red) and our projections (in black). We can see that WorldPop assumed a more aggressive population growth model than actually occurred.

| Multivariate model with worldpop data | | | With our population projections | |
| --- | --- | --- | --- | --- |
|  | Province level | District level | Province level | District level |
| Time to  vaccination center (1h) | 0.259  (0.255 to 0.263) | 0.182  (0.178 to 0.185) | 0.265  (0.261 to 0.279) | 0.196  (0.192 to 0.199) |
| Time to  provincial capital (1h) | 2.523  (2.427 to 2.624) | 2.685  (2.582 to 2.792) | 2.334  (2.244 to 2.426) | 2.934  (2.824 to 3.049) |
| Urban  proportion (10%) | 1.097  (1.095 to 1.099) | 0.991  (0.989 to 0.993) | 1.092  (1.090 to 1.094) | 1.017  (1.015 to 1.019) |

Line 216-218: “The accessibility rasters were aggregated for each province and district polygon by extracting the median travel time from patient residence province or district to PEP center or provincial capital value.”

This sentence is a bit hard to follow, I would suggest rewriting to: The accessibility measure was aggregated by means of extracting the median travel time per province or district. This could ultimately be linked to the patient’s record (i.e. the district or province of residence).

Modified as suggested

Paragraph 2.2.1: I would advise you to use a constrained (using building footprints) gridded population dataset as input data (https://www.worldpop.org/methods/top_down_constrained_vs_unconstrained). It allows you to have more accurate information on the actual location of population and thus the population living beyond the travel time catchments you are indicating (i.e 60 and 120 min). The calculations you make involve two aggregated measures (namely aggregated travel time and aggregated population counts) while in fact you have finer scale spatial data available to deal with the calculations in a more precise manner.

This would indeed be ideal but as discussed above, since WorldPop estimates relied a data from no more recent than 2008, we felt that projections by 2016 (the year in which we did our comparison) had deviated to far from the actual population totals and distribution. Thus the gain in resolution would result in a loss in accuracy.

Paragraph 2.2.3: This paragraph is not very clear to me and my feedback links back to the more general point raised before “to consider merging the estimates of dogbite patients and the estimates of positive animal samples”. To me it does not seem logical to predict the number of dogs that would be brought in for testing. This would make sense if your goal is to scale up your FAT testing capacity, but not to predict the risk of rabid dogbites. In my opinion it would make more sense if you use the number of positively tested samples among the patients that brought in a sample, as a proxy for the number of rabid dog bites and thus potentially averted deaths by the use of PEP. In a recent article written by Rajeev et al (2021), dogbites in Madagascar were used in a decision tree model to estimate the risk of a rabid dogbite in a Bayesian MCMC model. If this is also your aim, I would consider redoing a part of your model here and re-describe your goal and method here.

Following coments from reviewer we decided to remove this section of the analysis as the main goal of this paper focused on PEP patient accessibility. Testing will be covered in a future paper.

Line 252-254: Sentence “In an aggregated logistic regression..” is a bit challenging to read I would suggest to rewrite.
Section removed
--------------------

**Results**


-Does the analysis presented match the analysis plan?

-Are the results clearly and completely presented?

-Are the figures (Tables, Images) of sufficient quality for clarity?


Reviewer #1: (No Response)

Reviewer #2: Results
The results presented reflect the methods introduced before and are clearly lined out in text and figures. However, I find paragraph 3.3. challenging and would suggest to restructure and rewrite this. Below you can find a more detailed description.

Line 279-280: Were all these patient reports complete? How many were available at the province level and how many at the district level?

Incomplete record numbers added.

Paragraph 3.2: For the statistics about access to PEP I would highly suggest you to use a gridded population dataset instead of the aggregated counts. See example Rajeev et al. (2021). This would really increase the validity of your estimates. Proportionally calculating people’s access to care without taking into account their exact location leads to too much uncertainty of your estimates.

As discussed above, after comparing data sources, we prioritized accuracy over resolution as WorldPop data are not measured data but are also estimates and projections using the same primary source of measured data that we used: the Cambodia census which is published at the Provincial, District and Commune level. However we relied on the 1998, 2008 and 2019 censuses as opposed to only the two first ones for WorldPop, leading to inaccuracies in projections post-2008, with most of our modelling focusing on the years 2013 to 2016.

Line 355-356: Can you compare the provincial and district models? Since not all input data was collected at the district level, I would also expect a less powerful model output on district level because not all bite reports can be brought back to this level.

Indeed the two models are not directly comparable due to the difference in spatial scale and sample size. This was seen as a simple but not ideal approach to compare model fit.

Paragraph 3.3: It’s a well written paragraph but it remains unclear to me what exactly you want to model and to communicate to your audience. I think the main goal is to understand the distribution of rabies risk and thus the need to improve the spatial availability of PEP in high risk regions. To me it seems therefore that you would like to report 1) on the number of patients estimated to have missed out on PEP because of poor access, which would be your predicted number of patients minus the reported number of animal bite patients 2) the estimated risk of a patient being bitten by a rabid animal, which would be the probability of an animal to be rabid times the number of reported animal bites.

The increase in PEP patients to me seems a bit as an indirect measure. Because it represents the estimated total number of patients based on access to PEP centers over the last years. So reporting onthe regions where the difference between reported and predicted bite victims is highest would be the most important outcome, that is where you are missing access.

Indeed, due to the lack of direct measure of rabies in humans and dogs in Cambodia, accessibility is an indirect method to attempt to identify underserved areas. The specifics of this paper can’t really identify high-risk areas in that no measure of actual rabies is included. The different scenarios were used to estimate point 1) and identify where opening a new center would close that gap the most. A second paper focusing on tested animals will be used to infer geographical risk of exposure amongst other predictors of positive rabies animals and will answer point 2).

--------------------

**Conclusions**


-Are the conclusions supported by the data presented?

-Are the limitations of analysis clearly described?

-Do the authors discuss how these data can be helpful to advance our understanding of the topic under study?

-Is public health relevance addressed?


Reviewer #1: 6) In either the introduction/rationale or discussion/future work it would be helpful to understand whether building new treatment centers is the best way to prevent deaths due to rabies, compared with using those funds to make treatments free, increase awareness, vaccinate dogs, or some combination thereof, and how you might integrate that information with the results from this type of analysis.

A paragraph was added just before the conclusion addressing the limitations of the current strategies implemented in Cambodia, and current and future efforts to expand these.

Reviewer #2: Discussion & conclusion
Again, well written and provides a good overview of the results but would be more tangible with reports on measures as “averted deaths”, “human burden of diseases”, “proportion of rabid positive bites”

Line 431: “bite injuries” instead of “bites injuries”

corrected

Paragraph 4.2: The authors mention an important limitation of the accessibility layer used in paragraph 4.4, reflecting the static usage of the layer which is likely to have changed over the years because of improvements in the road network. However, the way accessibility is measured in this study brings another uncertainty to the estimates. The widely used malaria atlas friction layer makes large scale assumptions on road networks and travel speeds on different landcover types, which in fact might be different for your target population, namely people who seek access to care. In the paper published by Weiss et al (2018) https://www.nature.com/articles/nature25181. You can find back the travel speeds applied to each country. I would highly suggest you to have a look and check whether they seem reliable for your target population.

A section in the limitations regarding this was added.
--------------------

**Editorial and Data Presentation Modifications?**


Use this section for editorial suggestions as well as relatively minor modifications of existing data that would enhance clarity. If the only modifications needed are minor and/or editorial, you may wish to recommend “Minor Revision” or “Accept”.

Reviewer #1: - In the title, “prediction of future needs” is misleading since that is not actually what is done in this study, and I suggest removing or rephrasing.

Corrected as suggested
- In Fig 2, it would be helpful to see results in both absolute and relative terms since the numbers, especially since the overall are so small.

A new figure was added that includes absolute numbers by province and year
- If Fig 3b, the “missing” label seems incorrect.

As we are removing the dog testing section of this paper, this figure will be removed and used in our second paper.
- In Fig 4, please include a panel for scenario 4.

The fourth panel was added, and also to the accessibility raster image
- A lot of my comments require adding additional clarity or details. Since the manuscript is already long, some of the more descriptive text and discussion could be made more concise or removed.

Reviewer #2: Figures:
Overall great figures, but would suggest to clip the estimated travel times to the country borders.

Corrected

--------------------

**Summary and General Comments**


Use this section to provide overall comments, discuss strengths/weaknesses of the study, novelty, significance, general execution and scholarship. You may also include additional comments for the author, including concerns about dual publication, research ethics, or publication ethics. If requesting major revision, please articulate the new experiments that are needed.

Reviewer #1: (No Response)

Reviewer #2: Thank you for the opportunity to review this interesting paper on the effect of accessibility to PEP providing health centers on bite patient estimates to support rabies control strategies. The authors have written a very good piece and the scope of the work fits well with the PLoS NTD audience. Rabies research faces a lot of challenges regarding data availability and the authors applied thorough methods to estimate the number of actual bite victims in comparison to the reported bite victims in Cambodia. However, some of the methods and results have to be adapted to further improve the validity of the outcomes.

My main concerns are related to the population data in combination with the accessibility measures. Aggregated population counts in combination with aggregated measures of accessibility, create more uncertainty in the predictions and outcomes mentioned in the results. Since the travel time rasters are gridded I would highly suggest the authors to also use a gridded population dataset that provides information on the location of the population, like the WorldPop constrained dataset.

In addition, the way the results are written make it hard to follow the actual goal of the paper, which in my opinion is to estimate the risk of a rabid animal bite. I would consider a stronger emphasis on the number of patients estimated to be missed by the PEP clinics (estimated patients – reported patients) and the number of estimated rabid positive bites (number of positive samples * estimated animal bites). A visual representing the aimed outcomes or a decision tree model would maybe help guide the reader in understanding the results better. I provide more detail below.

The paper would also greatly benefit from a detailed read through to improve grammar. There are quite some inconsistencies with missing words or words that are plural and miss and s at the end.

Finally, the accessibility measures that are adapted from the global friction surfaces as provided by the Malaria project introduce uncertainty on actual travel times of the target population, because speeds on roads and landcover types have been set-up in a global studuy, not always reflecting the local context realistically.

Yet, the paper provides useful insights into an important NTD in a context that lacks recent research. The paper has high potential for publication when the feedback provided is taken into consideration.

All the best with the suggestions and I’m looking forward to reading an improved version of the paper!

--------------------

PLOS authors have the option to publish the peer review history of their article ([what does this mean?](https://journals.plos.org/plosntds/s/editorial-and-peer-review-process#loc-peer-review-history)). If published, this will include your full peer review and any attached files.


If you choose “no”, your identity will remain anonymous but your review may still be made public.


**Do you want your identity to be public for this peer review?** For information about this choice, including consent withdrawal, please see our [Privacy Policy](https://www.plos.org/privacy-policy).

Reviewer #1: No

Reviewer #2: No


Figure Files:
While revising your submission, please upload your figure files to the Preflight Analysis and Conversion Engine (PACE) digital diagnostic tool, https://pacev2.apexcovantage.com. PACE helps ensure that figures meet PLOS requirements. To use PACE, you must first register as a user. Then, login and navigate to the UPLOAD tab, where you will find detailed instructions on how to use the tool. If you encounter any issues or have any questions when using PACE, please email us at figures@plos.org.
 
Data Requirements:
Please note that, as a condition of publication, PLOS' data policy requires that you make available all data used to draw the conclusions outlined in your manuscript. Data must be deposited in an appropriate repository, included within the body of the manuscript, or uploaded as supporting information. This includes all numerical values that were used to generate graphs, histograms etc.. For an example see here: http://www.plosbiology.org/article/info%3Adoi%2F10.1371%2Fjournal.pbio.1001908#s5.
 
Reproducibility:
To enhance the reproducibility of your results, we recommend that you deposit your laboratory protocols in protocols.io, where a protocol can be assigned its own identifier (DOI) such that it can be cited independently in the future. Additionally, PLOS ONE offers an option to publish peer-reviewed clinical study protocols. Read more information on sharing protocols at https://plos.org/protocols?utm_medium=editorial-email&utm_source=authorletters&utm_campaign=protocols

__________________________________________________
In compliance with data protection regulations, you may request that we remove your personal registration details at any time. (Use the following URL: https://www.editorialmanager.com/pntd/login.asp?a=r). Please contact the publication office if you have any questions.
