# Supplementary material for: Accessibility to rabies centers and human rabies post-exposure prophylaxis rates in Cambodia: A Bayesian spatio-temporal analysis to identify optimal locations for future centers
Source: PLoS Negl Trop Dis. 2022 Jun 30;16(6):e0010494. doi: 10.1371/journal.pntd.0010494 (PMC9491732; doi:10.1371/journal.pntd.0010494)
Supplement: Attachment — Submitted filename: Responses to reviewers 2.docx [file pntd.0010494.s013.docx]

| UNIVERSITY OF CALIFORNIA, DAVIS | |  |  | |
| --- | --- | --- | --- | --- |
| BERKELEY • DAVIS • IRVINE • LOS ANGELES • MERCED • RIVERSIDE • SAN DIEGO • SAN FRANCISCO | |  | SANTA BARBARA • SANTA CRUZ | |
| UNIVERSITY OF CALIFORNIA  Center for Animal Disease Modeling and Surveillance (CADMS)  Department of Medicine & Epidemiology  School of Veterinary Medicine  Phone: (530) 752-7675  Fax: (530) 752-0414 | ONE SHIELDS AVENUE  DAVIS, CALIFORNIA 95616-8734 | | |  |

Uppsala, Sweden, February 25, 2022

Dear editors and reviewers

I thank you again for the comments and suggestions offered throughout the different steps of this review process. Please find attached our responses to our manuscript entitled “Accessibility to rabies centers and human rabies post-exposure prophylaxis rates in Cambodia: a Bayesian spatio-temporal analysis to identify optimal locations for future centers”. We hope to have addresses all issues highlighted by the reviewers as to make this manuscript suitable for publication.

Thank you in advance for your consideration,

Best Regards,

Jerome Baron, DVM, MSc

Center for Animal Disease Modeling and Surveillance (CADMS)

Department of Medicine & Epidemiology

School of Veterinary Medicine

University of California

Davis, CA 95616 USA

jnbaron@ucdavis.edu

Section for Epidemiological Methods

Department of Epidemiology and Disease Control
National Veterinary Institute of Sweden

Uppsala, 75189 Sweden

jerome.baron@sva.se

**Methods**

Reviewer #1: I thank the authors for adding Table 1 in response to my comments. It is very helpful, but I suggest editing the text for "scenario 5" to reflect the description in the text of the methods because it's currently unclear. For example, the description could read something like: "21 scenarios with four PEP centers, where each scenario includes the three current PEP centers (Phnom Penh, Battambang and Kampong Cham) and one additional center added in each of the 21 provincial capitals." In addition, I'm unsure what "as scenario 1,2, and 3" means for the Source.

Corrected as suggested

Lines 314-315. "The result of this analysis was not significant". Please be more specific about what you were specifically testing here, and what was "not significant".

Sentence was reworded to clarify that the association between seasonality and PEP rates was not siginificant

Reviewer #2: Thank you very much for the opportunity to review a revised version of this manuscript. The paper was already very interesting and good at the first round of review, but the authors did an outstanding job at further improving the study. I would like to thank the authors for their detailed feedback on the comments raised and have no additional technical comments for consideration in this new round of revisions. The authors improved the explanation of the different scenarios substantially by adding an extra table and also further highlighted the limitations of the input data. I find the paper fit for publication after some small improvements of grammar and spelling. I think the paper would even further improve after a native english speaker could read over it.

**Results**
Reviewer #1: I'm a bit confused about why Scenario 5 gets introduced first in section 3.2. Shouldn't lines 460 to 471 belong in the last paragraph of the results where Table ST4 is referenced? If I am missing something here then it would be helpful to have further description of the rationale of this scenario in both sections of the results.

Results presented in paragraph 3.2 refer to the methods from paragraph 2.2.1 where use of the scenarios is stated. This section does not involve the prediction from the regression models and their prediction results. Section 3.4 refers to the regression model predictions (methods 2.2.2). However, table ST4 should have also been presented in section 3.2 given the results it contains. Table numbering, organization and caption have been changed to reflect this issue.

Reviewer #2: Yes

**Conclusions**
Reviewer #1: Their conclusions are supported and in their revisions the authors have added additional limitations and public health context important for understanding the implications.

Reviewer #2: Yes

**Editorial and Data Presentation Modifications?**
Reviewer #1: The title of the manuscript is still a bit misleading. In this paper, the authors examine associations between accessibility and yearly PEP patient rates (and use that fitted relationship to predict optimal new PEP center locations), rather than causally assessing the "impact". I suggest replacing "impact" with "association between". Alternatively, the title could be rephrased to read something along the lines of: "Accessibility to rabies centers and human Rabies post-exposure prophylaxis rates in Cambodia: a spatio-temporal analysis to identify optimal locations for future centers".

The title has been change as per the reviewer’s suggestions.

Reviewer #2: I think the paper would even further improve by having a native speaker go over the text once. I started to notice some tiny mistakes in the abstract (see below), but then focussed more on the bigger picture of the paper and the content.

Title
“in Cambodia and identification of optimal future center locationsusing spatio-temporal Bayesian regression modelling” add space between locations & using.

Abstract

Line 34: “to inform the selection future PEP center locations in high-risk areas” suggest to change sentence to “to inform the selection of future PEP center locations in high-risk areas”

Line 35: “accessibility to rabies centers on the yearly rate of PEP patient in the population and generate a risk map” suggest to add and “s” behind patient > PEP patients

Line 38: “patients as outcomes” remove “s” behind outcome.

Line 39: “Secondary exposure variables consisted in” change to “consisted of”

A complete re-read of the manuscript was undertaken as suggested
--------------------

**Summary and General Comments**
Reviewer #1: Overall, the authors have done a thorough job of responding to my comments and my remaining comments are mostly editorial.

Reviewer #2: Strong study with a very important societal impact in a rabies endemic country. Authors have been able to produce reliable estimates of human rabies and dogbite burden that can aid prioritization and allocation of resources. Figures and tables support the findings of the studies and the improvements made by the authors help guide the reader through the paper. Well done and I look forward to seeing the rest of the research coming alive!

We thank the reviewers for their thoughtful suggestions throughout this review.
